# High expression of miR-493-5p positively correlates with clinical prognosis of non small cell lung cancer by targeting oncogene ITGB1

**DOI:** 10.18632/oncotarget.17650

**Published:** 2017-05-07

**Authors:** Zhu Liang, Rui Kong, Zhan He, Li-Yao Lin, Shan-Shan Qin, Chun-Yuan Chen, Zhan-Qiang Xie, Fei Yu, Guo-Qian Sun, Chun-Guang Li, Da Fu, Geng-Xi Jiang, Jie Chen, Yu-Shui Ma

**Affiliations:** ^1^ Department of Cardiothoracic Surgery, The Affiliated Hospital of Guangdong Medical University, Zhanjiang 524001, China; ^2^ Medical College of Soochow University, Soochow 215006, China; ^3^ Department of Nuclear Medicine, Shanghai Tenth People's Hospital, Tongji University School of Medicine, Shanghai 200072, China; ^4^ Department of Thoracic Surgery, Changhai Hospital of Second Military Medical University, Shanghai 200433, China; ^5^ Shanghai Engineering Research Center of Molecular Therapeutics and New Drug Development, College of Chemistry and Molecular Engineering, East China Normal University, Shanghai 200062, China

**Keywords:** ITGB1, miR-493-5p, biomarker, survival, target

## Abstract

Increasing evidence supports that microRNA (miRNA)-mediated gene regulation plays a significant functional role in cancer progression. To investigate the expression and clinical significance of ITGB1 in non small cell lung cancer (NSCLC), the expression levels of ITGB1 in NSCLC tissues and human normal lung tissues were analyzed in silico using genes microarray, KEGG pathway and hierarchical clustering analysis followed by validation with quantitative RT-PCR. Our results showed that ITGB1 was upregulated in NSCLC tissues when compared with normal lung tissues. Survival analysis based on the qRT-PCR data established that ITGB1 expression was attentively related to the prognosis of NSCLC, and patients with higher ITGB1 expression had shorter overall survival (OS). Moreover, ITGB1 was confirmed to be a direct target of miR-493-5p. Furthermore, concomitant high expression of ITGB1 and low expression of miR-493-5p correlated with a shorter median OS and PFS in NSCLC patients. In conclusion, our results provide the first evidence that ITGB1 is a direct target of miR-493-5p suggesting that ITGB1 and miR-493-5p may have potential prognostic value and may be useful as tumor biomarkers for the diagnosis of NSCLC patients.

## INTRODUCTION

Lung cancer is the third peak frequent human malignant tumor and the leading cause of cancer death in worldwide [1], among which non–small cell lung cancer (NSCLC) accounts for 75 to 80% of lung cancer incidents [2] and are mostly diagnosed at late stage. Recently, although the emergency of fresh drugs and therapeutic schedules, the prognosis of lung cancer is still poor and 5-year overall survival rate is only about 11% [3]. Morever, current staging approaches are inadequate in predicting and diagnosing the outcome of NSCLC treatments due to the unavailability of potential biomarkers for molecular targeted or personalized treatments [4]. Therefore, improvement in molecular genetics diagnosis and the prediction of prognosis for targeted treatments and clinical decisions are urgently required.

MicroRNAs (miRNAs) are a class of small non-coding RNAs (ncRNAs) play a key role in the regulation of mRNA translation and degradation through base pairing to a partially complementary site, predominantly in the untranslated region (UTR) of the target messenger RNAs (mRNAs) [5]. miRNAs can posttranscriptionally regulate the expression of hundreds of their target genes, thereby controlling a wide range of biological functions such as cellular proliferation, differentiation, development and apoptosis [6]. Dysfunctional regulation of miRNAs in several ailments has been associated with a range of diseases, including cancers [7]. The miRNAs have the potential to regulate the expression of thousands of corresponding target genes, and are able to govern a comprehensive range of biological functions such as cellular proliferation, differentiation, apoptosis, immune responses, and the maintenance of cell and tissue identity. Therefore, the results of molecular exploration in miRNAs may improve the clinical decisions and management for NSCLC patients and resulte in generation of many candidate non-invasive biomarkers with potential clinical significance.

Previous studies have identified various miRNAs in NSCLC patients, including Let-7a, miR-145-3p, miR-30d, miR-499, miR-374a, miR-1254, miR-574-5p, miR-126, miR-210, miR-21 and so on [8]. There has previously reported that low levels of miR-33a expression were found in NSCLC patients in clinical and suggested that the *miR-33* family might play a significant role in NSCLC prognosis and patient survival [9]. Recent reports have suggested that down-regulation of miR-138, miR-218, miR-34c-3p were found in NSCLC [10–12]. Therefore, miRNAs play an important role in diagnosis, prediction and treatment of NSCLC as a new symbol of molecular biology, which becoming one of the highlights in agro-scientific research in the life sciences.

However, there has no previous report that investigate the correlation between the expression level of miR-493-5p and target gene ITGB1 in NSCLC. In this study, we profiled miRNAs and genes expression by microarray to identify their differentially expression in NSCLC and adjacent normal tissues, and then explore the correlation between miR-493-5p and ITGB1 in NSCLC, which will help to give further insight into the pathogenesis of the NSCLC.

## RESULTS

### Differential gene expression analysis using GEO database

**Figure 1:**
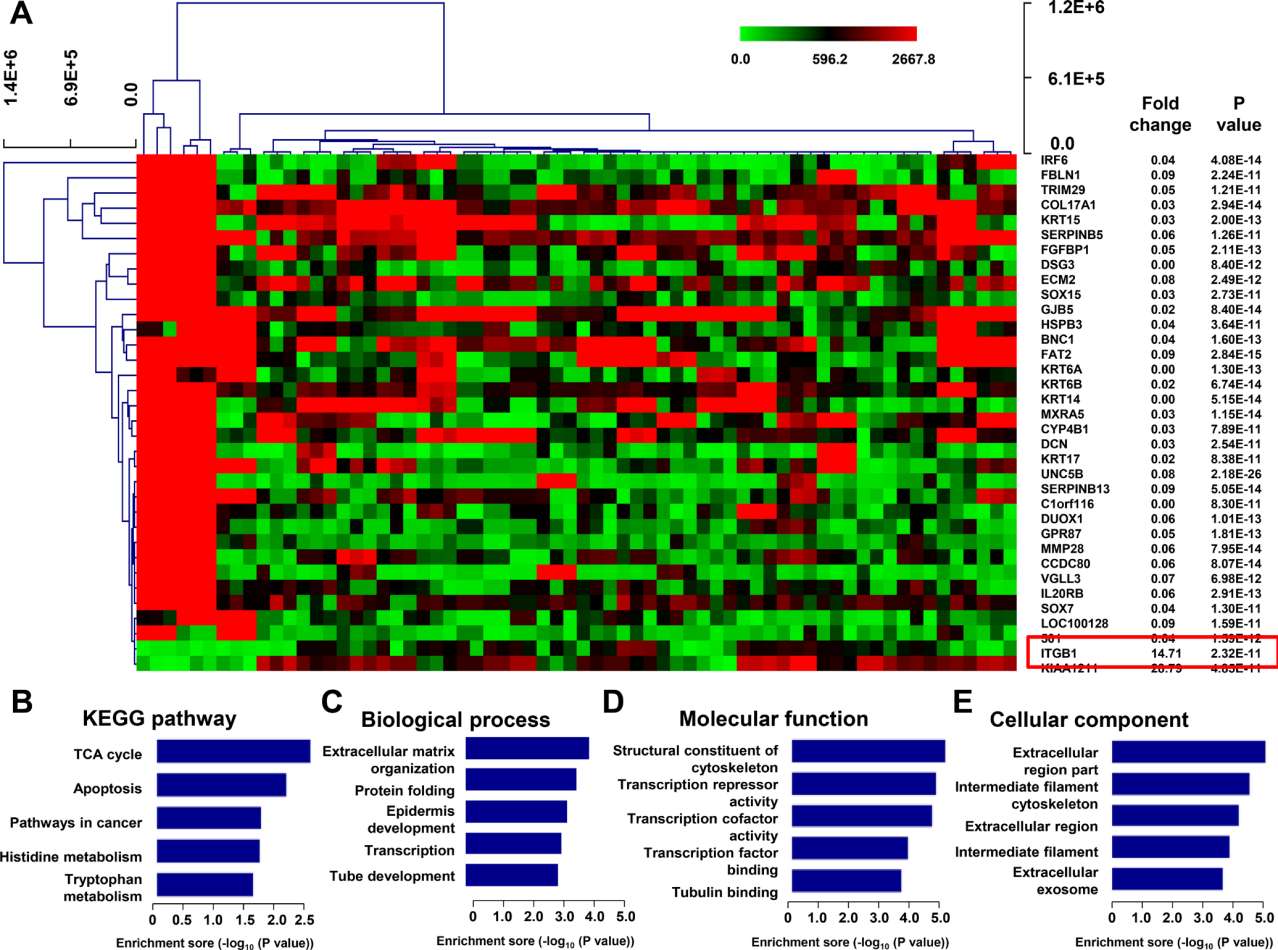
Differential gene expression in NSCLC using the GEO datasets (**A**) Clustered analysis of differential expression of mRNAs (Fold change ≥10 or ≤ 0.1, *P* < 1E-10) in GSE41445 from GEO database including 18 cancer cells and 3 non-tumorigenic cell lines. A total of 34 differential genes were found, including ITGB1, which indicated by red box. KEGG pathway analysis (**B**) and GO analysis of 34 differentially expressed genes associated with biological process (**C**), molecular function (**D**) and cellular component (**E**).

To investigate the general disordered genes in tumor, we downloaded one group of data from GEO database (GSE41445) which included gene expression data of 18 cancer cells and 3 non-tumorigenic cell lines. We found 2 upregulated and 32 downregulated mRNAs (fold change (FC) ≥ 10 or ≤ 0.1, *P* < 1E-10) in cancer cells when compared with normal cell lines (Figure 1A).

### Kyoto encyclopedia of genes and genomes (KEGG) pathway analysis

To elucidate the key pathways involveld of the 34 differentially expressed transcripts in malignant cancer cells, we executed KEGG pathways analysis and revealed many enrichment-related pathways including TCA cycle, Apoptosis, Pathways in cancer, Histidine metabolism, and Tryptophan metabolism (Figure 1B).

### Gene ontology (GO) analysis

To elucidate the relationship between gene differential expression patterns in normal and malignant cancer cells, we examined the functional bias of the 34 differentially expressed transcripts according to GO classifications. These differentially expressed transcripts were grouped into 25 GO based on biological process GO terms. The most enriched GO terms (*P* < 1E-3) included extracellular matrix organization, protein folding, epidermis development, transcription, and tube development, arguing that the extracellular signaling connecting tumor and stromal cells was vital to regulate cancer cell malignant phenotypes (Figure 1C).

Molecular function analysis showed that the differential genes were significantly enriched with those related to protein transcription, such as transcription repressor activity, transcription cofactor activity, transcription factor binding. Interestingly, the most significantly enriched molecular functions of these differential genes was structural constituent of cytoskeleton, which involved in molecules that contributes to the structural integrity of a cytoskeletal structure (Figure 1D).

We further analyzed the subcellular localization of the identified differentially expressed genes. Similarly, cellular component analysis showed that the differential genes were significantly enriched with those related to extracellular region and cytoskeleton, such as extracellular region part, extracellular region, intermediate filament, extracellular exosome, and intermediate filament cytoskeleton (Figure 1E).

Among the upregulated genes, ITGB1, an integral membrane protein forming a receptor for many extracellular-matrix proteins, had an FC score of 14.71 (*P* = 2.32E-11) (Figure 1A) and aroused our great interest in studying its role in promoting NSCLC tumorigenesis.

### The expression levels of ITGB1 in tumor cell lines

**Figure 2:**
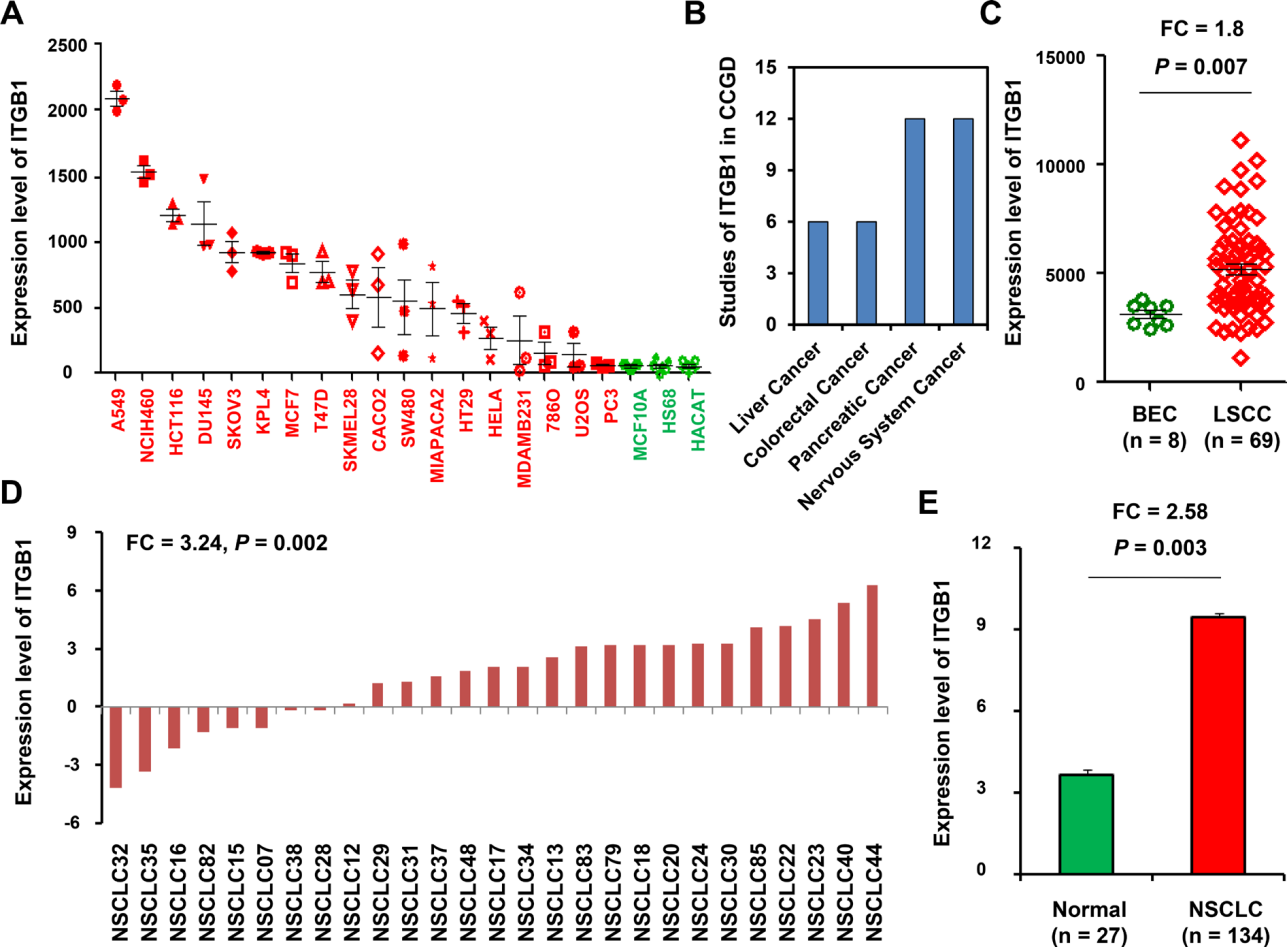
Analysis of ITGB1 expression in NSCLC (**A**) Relative expression levels of ITGB1 in different cancer cell lines *vs*. normal controls from the GEO dataset (GSE41445). (**B**) ITGB1 was indicated as a potential cancer driver in the Candidate Cancer Gene Database (CCGD), involved in liver, colorectal, pancreatic and nervous system cancer. (**C**) Relative expression levels of ITGB1 in a microarray profiles data (GSE67061-GPL6480) from GEO database, which including 8 cases of immortalized human bronchial epithelial cell lines (BEC) and 69 cases of lung squamous cell carcinoma tissues (LSCC). (**D**) ITGB1 expression levels in cancer *vs*. paired adjacent non-tumor tissue (*n* = 27). (**E**) ITGB1 expression in tumor samples (*n* = 134) and adjacent non-tumor lung tissue (*n* = 27) were analyzed using qRT-PCR assay.

We analyzed the expression levels of ITGB1 in data of GSE41445 and found that ITGB1 had the highest expression level in two NSCLC cell lines (incluing lung adenocarcinoma cell A549 and large cell lung cancer cell NCI-H460) (Figure 2A), suggesting that ITGB1 may play an important role in tumorigenesis and progress of lung cancer.

Previously, there were 24 studies indicated that ITGB1 was a potential cancer driver in the Candidate Cancer Gene Database (CCGD), involved in liver, colorectal, pancreatic and nervous system cancer (Figure 2B). We next explore the expression levels of ITGB1 using genes microarray data followed by validation with quantitative RT-PCR in NSCLC specimens and normal lung tissues.

### Expression of ITGB1 between NSCLC and normal lung tissues

Next, we downloaded a microarray profiles data (GSE67061-GPL6480) from GEO database, which including 8 cases of immortalized human bronchial epithelial cell lines (BEC) and 69 cases of lung squamous cell carcinoma tissues (LSCC) and found that expression of ITGB1 was also significantly increased in LSCC when compared with that in BEC controls (FC = 1.8, *P* = 0.007; Figure 2C).

We then validated the expression levels of ITGB1 in NSCLC tissue samples using qRT-PCR assay in 134 NSCLC biopsies, 27 of which were pairs of tissue from para-carcinoma tissues. In 27 paired of NSCLC samples, ITGB1 was highly expressed in lung cancer relative to non tumor samples via qRT-PCR analysis (FC = 3.24, *P* = 0.002; Figure 2D).

In order to validate whether ITGB1 expression levels were affected by clinical characteristics, we explored the correlation of ITGB1 expression with demographic and clinical factors. All 134 patients of NSCLC included in this study demonstrated that ITGB1 expression levels were positively correlated with lymph-node metastasis (*P* = 0.036), TNM stage (*P* = 0.042) and the diameter of the tumor (*P* = 0.044) as shown in Table 1. However, we did not observe any association between ITGB1 expression and patient age, gender, smoking history, tumor differentiation and histology (*P* > 0.05, Table 1).

**Table 1:** Univariate analysis of overall survival in NSCLC patients stratified based on clinical characteristics

Factor	Variable	*N*	*ITGB1* expression (Median)	*P* value	Overall survival		
					Months (Median)	95% CI (Mean)	*P* value (Log - rank test)
Age							
	≥ 60	92	29.76	0.228	30.05	26.72–34.26	0.433
	< 60	42	21.53		32.43	29.21–35.93	
Gender							
	Male	76	23.38	0.921	32.11	29.22–34.39	0.526
	Female	58	22.77		33.67	30.05–35.79	
Smoking history							
	Never	34	24.34	0.723	32.21	28.46–34.17	0.129
	Ever	46	23.65		30.96	27.65–33.18	
	Unknown	54	26.47		30.63	25.36–33.68	
Lymph-node metastasis							
	Negative	82	18.74	**0.036**	32.73	28.72–33.68	**0.038**
	Positive	43	25.43		28.01	26.54–30.45	
	Unknown	9	18.96		30.28	28.34–33.02	
Tumor differentiation							
	Poorly	51	25.12	0.052	28.64	26.57–31.43	0.158
	Moderately	78	19.96		29.38	27.08–32.66	
	Well	5	20.08		31.61	29.35–33.79	
Histology							
	Adenocarcinoma	72	24.67	0.658	29.54	27.81–31.99	0.079
	Squamous cell carcinoma	60	26.03		31.66	28.46–32.83	
TNM stage							
	I–II	94	25.64	**0.042**	33.58	30.17–35.38	**0.012**
	III–IV	40	29.87		27.63	24.75–29.68	
Diameter							
	≥ 5 cm	37	28.03	**0.044**	24.89	22.46–28.01	**< 0.001**
	< 5 cm	97	24.68		31.53	27.43–34.69	

Moreover, the level of ITGB1 expression was also higher in all NSCLC tumor biopsies (9.43 ± 0.87) than those in normal lung tissues (3.66 ± 0.74). The differences were statistically significant (FC = 2.58, P= 0.003; Figure 2E).

### Prediction of miRNAs targeting to ITGB1

To investigate the miRNAs targeting to ITGB1, we used three target genes prediction websites (MIRDB, RNA22 and Targetscan) to forecast potential miRNAs which target to ITGB1. The result showed that there were 7 common predicted miRNAs, including miR-455-3p, miR-183-5p, miR-29c-3p, miR-29b-3p, miR-29a-3p miR-124-3p, and miR-493-5p. Of these 7 genes, miR-455-3p, miR-183-5p and 3 miR-29 family member have been reported [13–15] and validated to target ITGB1. The remaining two miRNAs, miR-124-3p and miR-493-5p, had not been previously reported to target and regulate ITGB1 in NSCLC. Therefore, we sought to further analyze the expression of miR-124-3p and miR-493-5p in NSCLC patients.

### Expressionof miR-493-5p in NSCLC and normal samples

**Figure 3:**
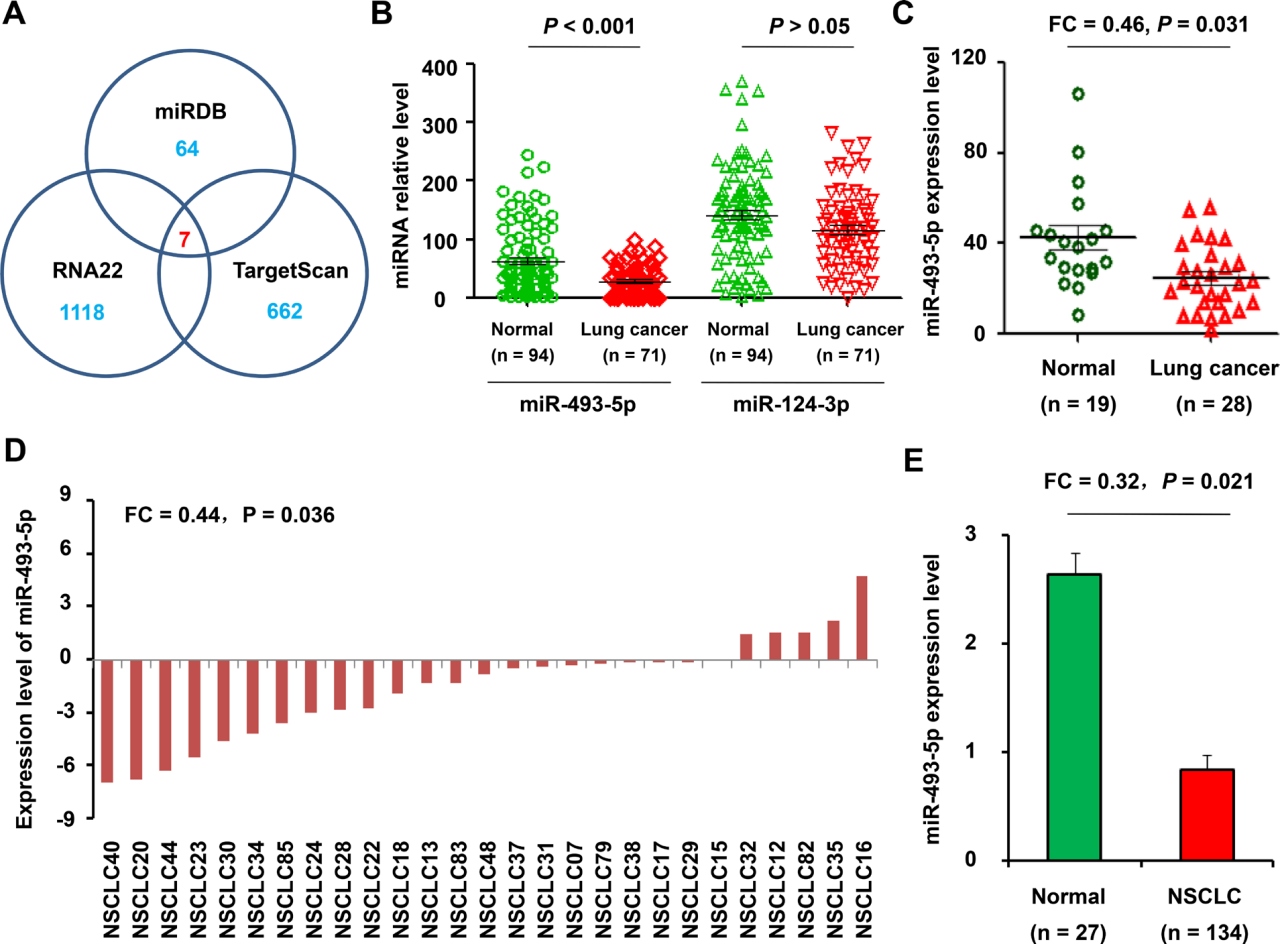
Expression of miR-493-5p in NSCLC and normal samples (**A**) miRNAs targeting to ITGB1 were predicted using three target genes online prediction programs (MIRDB, RNA22 and Targetscan). (**B**) Differential expression analysis of miR-124-3p and miR-493-5p in GSE61741 from GEO database including 71 lung cancer patients and 94 healthy individuals. (**C**) The levels of miR-493-5p in peripheral miRNA blood profiles (GSE24709) from 28 patients with lung cancerous and 19 normal controls were analyzed. (**D**) miR-493-5p expression levels in cancer *vs*. paired adjacent non-tumor tissue (*n* = 27). (**E**) miR-493-5p expression in tumor samples (*n* = 134) and adjacent non-tumor lung tissue (*n* = 27) were analyzed using qRT-PCR assay.

We downloaded a peripheral blood profiles data (GSE61741) from GEO database, which including 71 lung cancer patients and 94 healthy individuals and validated that the expression level of miR-493-5p was significantly down-regulated (FC = 0.49, *P* < 0.001) in peripheral blood samples of 71 lung cancer patients. However, the difference of miR-124-3p expression in peripheral blood of lung cancer patients was not statistically significant (FC = 0.81, *P* > 0.05) when compared with healthy individuals (Figure 3B).

Subsequently, peripheral miRNA blood profiles (GSE24709) from 28 patients with lung cancerous and 19 normal controls were also analyzed. When compared with normal controls, the expression level of miR-493-5p was significantly down-regulated (FC = 0.49, *P* = 0.031) in peripheral blood samples of 28 lung cancer patients (Figure 3C).

We further validated the expression levels of miR-493-5p in NSCLC tissue samples using qRT-PCR assay in 134 NSCLC biopsies, 27 of which were pairs of tissue from para-carcinoma tissues. In 27 paired of NSCLC samples, miR-493-5p was lowly expressed in lung cancer relative to non tumor samples (FC = 0.44, *P* = 0.036; Figure 3D). Moreover, the level of miR-493-5p expression was also lower in all NSCLC tumor biopsies (0.78 ± 0.19) than those in normal lung tissues (2.69 ± 0.21). The differences were statistically significant (FC = 0.32, *P* = 0.021) (Figure 3E).

### Validation of miR-493-5p/ITGB1 expression and correlation using qRT-PCR

**Figure 4:**
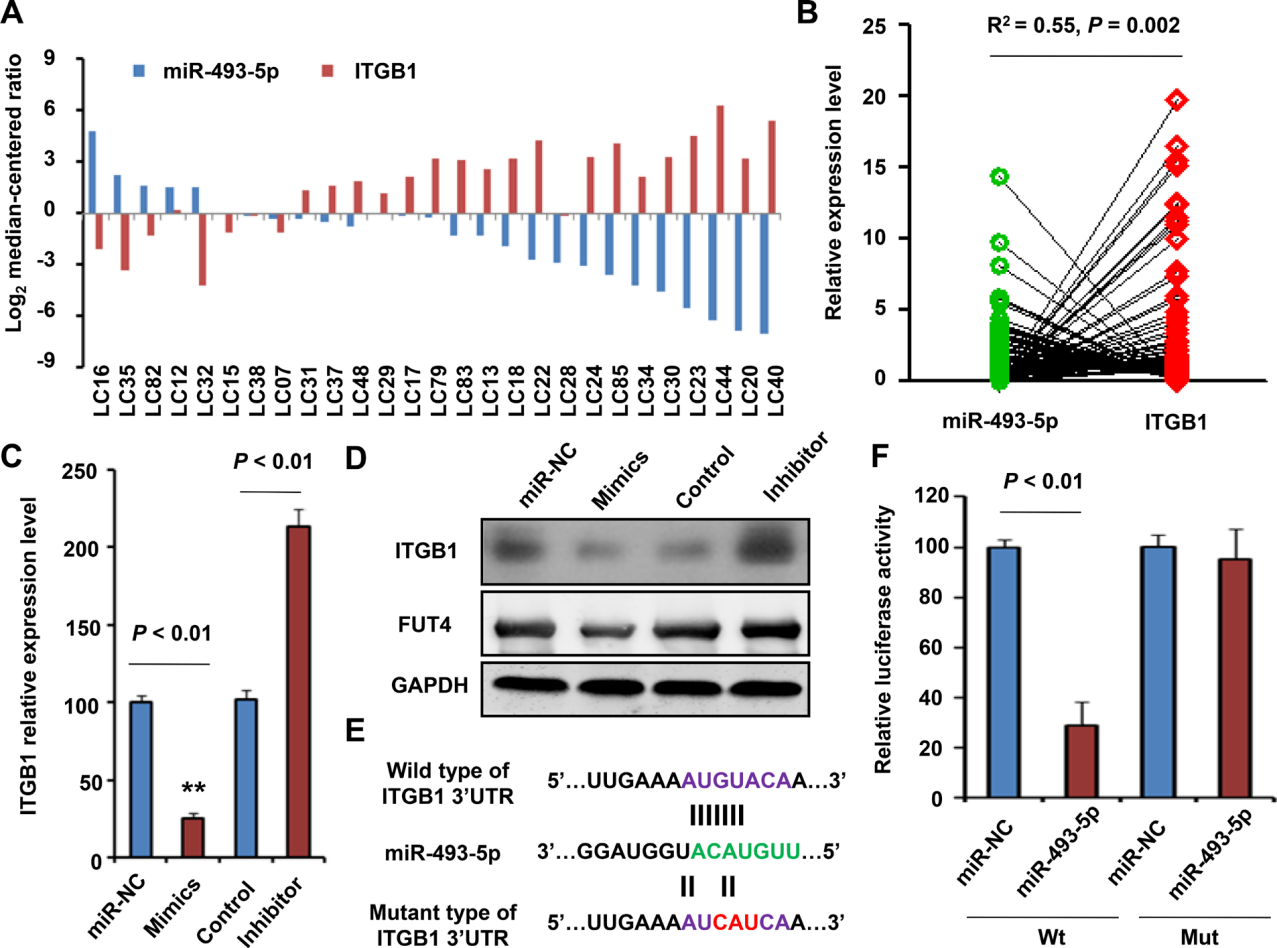
Validation of ITGB1 as a direct target of miR-455-3p (**A**) The relationship of ITGB1 levels with the expression of miR-493-5p in cancer *vs*. paired adjacent non-tumor tissue (*n* = 27). (**B**) The relationship of ITGB1 levels with the expression of miR-493-5p in 134 NSCLC samples. qRT-PCR (**C**) and Western blotting (**D**) were used to measure the mRNA and protein level of ITGB1 after treatment of miR-493-5p mimics (overexpression) or inhibitor (knockdown) in HeLa cells. (**E**) A schematic diagram of the miR-493-5p of 3′UTR in ITGB1 binding site and mutation site. (**F**) Luciferase activity assay of pGL3-ITGB1-3′UTR reporter co-transfected with miR-493-5p mimic or miR-493-5p -mut oligonucleotides in HeLa cells. Data shown are the means ± SD of three independent experiments.

To further verify the correlation between miR-493-5p and ITGB1, we analyzed the expression levels of miR-493-5p and ITGB1 in 134 NSCLC biopsies using qRT-PCR assay. Figure 4A showed that the expression level of miR-493-5p in 27 paired of NSCLC samples was lower than in normal lung tissues. However, ITGB1 over-expressed in NSCLC samples when compare with normal tissues, which imply that the expression level of miR-493-5p was negatively correlated with NSCLC samples in NSCLC.

Next we analyzed the correlation of miR-493-5p and ITGB1 in 134 NSCLC biopsies and found that the relative expression level between miR-493-5p and ITGB1 presented inverse correlation (R^2^ = 0.55, = 0.002; Figure 4B).

### Identification of ITGB1 as a target of miR-493-5p

To further confirm the relationship between miR-493-5p and ITGB1, we detected the ITGB1 mRNA and protein levels after treatment of miR-493-5p mimics or inhibitor in HeLa cells. When compared to control group, the expression level of ITGB1 was lower in miR-493-5p mimics group (*P* < 0.01) and was higher in the miR-493-5p inhibitor group (*P* < 0.01) (Figure 4C), which suggested that ITGB1 were regulated by and negatively correlated with miR-493-5p.

Then, to further test the regulation role of miR-493-5p on ITGB1 at the protein level, we used Western blotting to measure the levels of ITGB1 protein and a reported downstream protein FUT4 [16] after miR-493-5p overexpression or knockdown. The results suggested that the increase in miR-493-5p levels significantly decreased ITGB1 protein expression and had the same tendency in FUT4 and vice versa (Figure 4D).

Using bioinformatics analysis, we found that miR-493-5p contained specific binding sequence of the 3′-UTR region of *ITGB1* gene. The 3′-UTR binding site and mutation site of miR-493-5p of *ITGB1* gene are shown in Figure 4E. We performed a luciferase reporter assay to further verify whether miR-493-5p directly targeted *ITGB1*. As shown in Figure 4F, ectopic expression of miR-493-5p decreased the luciferase activity of the 3′-UTRs of *ITGB1*. However, miR-493-5p mutant containing three altered nucleotides in the seed sequence did not have an inhibitory effect on luciferase activity.

### Biological role of miR-493-5p and ITGB1 in NSCLC progression

**Figure 5:**
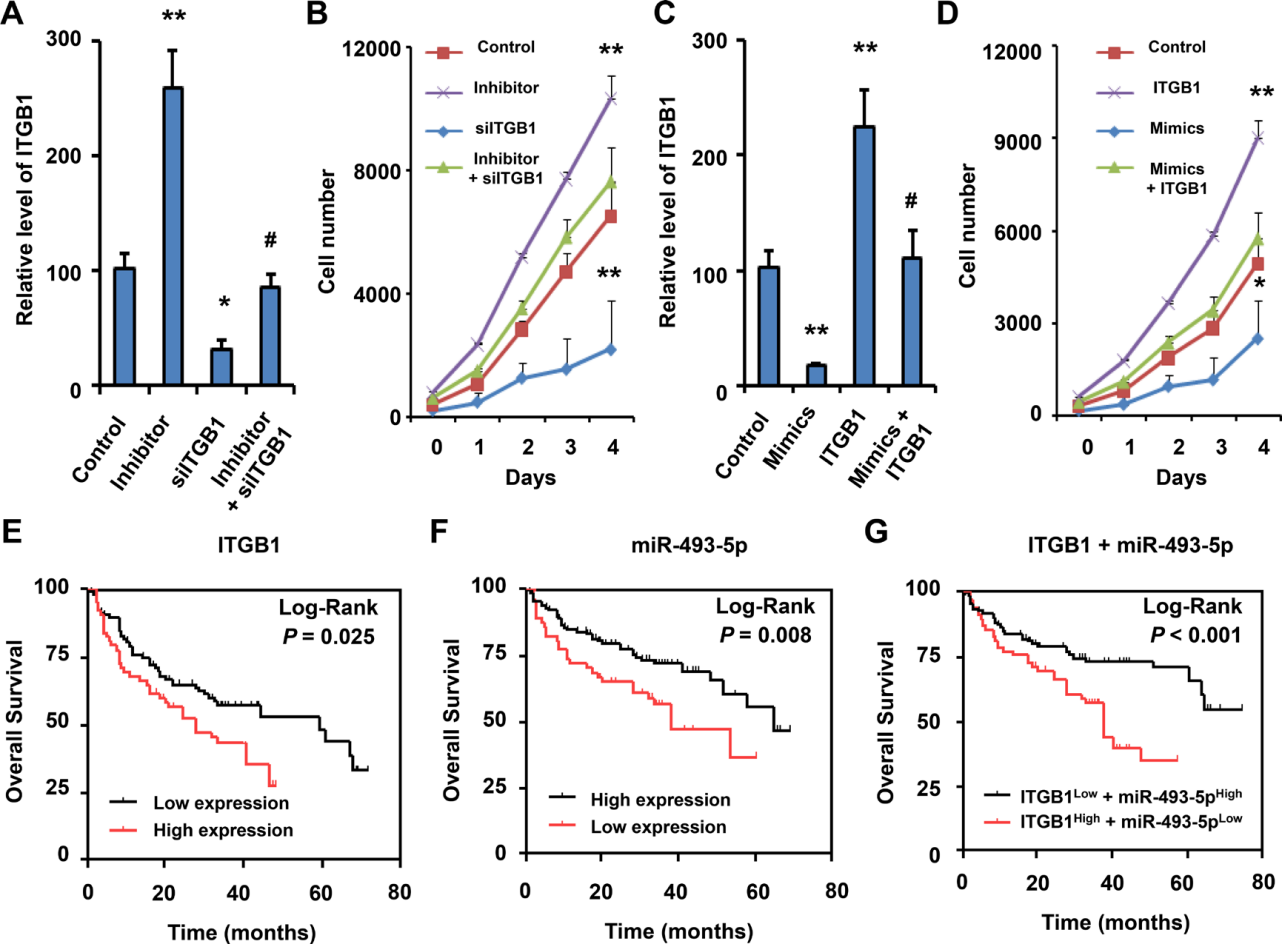
Biological role and clinical significance of miR-493-5p and ITGB1 in NSCLC (**A**) qRT-PCR measurement of the levels of ITGB1 mRNA in A549 cells treated with negative control, miR-493-5p inhibitor and/or siITGB1. Data shown are the means ± SD of three independent experiments. (**B**) The A549 cell counts in 96-well plate after transfection with negative control, miR-493-5p inhibitor and/or siITGB1 at the indicated day. (**C**) qRT-PCR measurement of the levels of ITGB1 mRNA in A549 cells treated with negative control, miR-493-5p mimics and/or ITGB1 expression vector. (**D**) A549 cell counts in 96-well plate after transfection with negative control, miR-493-5p mimics and/or ITGB1 expression vector at the indicated day. Data shown are the means ± SD of three independent experiments. Statistical analyses were performed with one-way ANOVA. Kaplan-Meier survival analysis was used to evaluate the prognostic value of ITGB1 (**E**) and miR-493-5p (**F**) expression in 134 NSCLC biopsies for OS analysis. (**G**) Kaplan-Meier survival analysis was used to evaluate the prognostic value of ITGB1 and miR-493-5p expression in 134 NSCLC biopsies for OS analysis (*P < 0.05; **P < 0.01; ^#^*P* > 0.05).

To investigate the biological roles of miR-493-5p and ITGB1 in NSCLC progression, we performed loss-of-function studies using a miR-493-5p inhibitor and siITGB1 on the NSCLC cell line A549 (Figure 5A). As shown in Figure 5B, suppression of miR-493-5p significantly enhanced the growth rate of A549 cells transfected with the miR-493-5p inhibitor compared with the negative control-transfected cells. However, cellular growth assay revealed that following siITGB1 transfection, the growth rate of A549 cells was significantly inhibited when compared to the control group. There was no a dramatic change in cellular growth between among the A549 cells with miR-493-5p inhibitor and siITGB1 transfection and negative control-transfected cells. Taken together, these results suggested that miR-493-5p downregulation promoted the proliferation of NSCLC cells and knockdown of ITGB1 inhibited NSCLC cell proliferation *in vitro*.

Next, we established the A549 cell line to stably express miR-493-5p, ITGB1, or vector (Figure 5C). The result of cellular growth assay showed that overexpression of miR-493-5p significantly inhibited the growth rate of A549 cells compared with control cells and overexpression of ITGB1 significantly promoted the growth rate of A549 cells (Figure 5D). There was no a dramatic change in cellular growth between A549 cells with miR-493-5p- and ITGB1-overexpression and A549 cells transfected with vector control. These results suggested that miR-493-5p upregulation inhibited NSCLC cell proliferative capacity and overexpression of ITGB1 promoted NSCLC cell proliferation *in vitro*.

### Clinical significance of miR-493-5p and ITGB1

Kaplan-Meier survival curves were plotted and log rank analysis was performed to evaluate the prognostic value of miR-493-5p and ITGB1 expression for patients with NSCLC. Our results indicated that ITGB1 expression was positively correlated with lower OS (*P* = 0.025) (Figure 5E) in NSCLC patients. Moreover, Kaplan-Meier analysis showed that patients with high miR-493-5p expression had longer OS compared to those with low miR-493-5p expression (*P* = 0.008) (Figure 5F).

Since the data had suggested that ITGB1 expression level was negatively correlated with miR-493-5p expression, and ITGB1 was a direct target of miR-493-5p, we further examined the prognostic value of ITGB1 expression together with miR-493-5p levels using multivariate analysis of OS by Kaplan-Meier survival analysis. The results showed that NSCLC patients with high ITGB1 expression and low miR-493-5p levels had significantly decreased OS (*P* < 0.001) (Figure 5G), which suggested that ITGB1 and miR-493-5p might have potential prognostic value and could be useful as tumor biomarkers for the diagnosis of NSCLC patients.

## DISCUSSION

NSCLC is one of the most lethal human cancers around the world and has increasing incidences with short survival rate and high mortality in reccent years. Therefore, the novel molecular mechanisms involved in the aggressive growth of NSCLC, and further new targeted therapies are highly required for prolonging the survival time of the lung cancer patients. The useful biomarkers that we need rely on their potential significant as a molecular targeted therapy in the treatment of NSCLC patients. In order to administrate and improve the current standard of clinical care, tremendous efforts have been taken to explore a molecular signature that would assist to the cancer diagnose and therapy.

Currently, a large number of studies have demonstrated that miRNA s are involved in the development of non-small cell lung cancer and increasing studies have confirmed that miRNA expression levels in NSCLC tissue *versus* normal lung tissue are not only statistically significant, but also correlate with NSCLC diagnosis, prognosis and treatment. Yanaihara et al. [17] examined the miRNA expression profiles for lung cancers to investigate miRNA's involvement in lung carcinogenesis and identified that high hsa-mir-155 and low hsa-let-7a-2 expression correlated with poor survival by univariate analysis as well as multivariate analysis for hsa-mir-155, indicating that miRNA expression profiles are diagnostic and prognostic markers of lung cancer. Yu et al. [18] investigated whether microRNA expression profiles can predict clinical outcome of NSCLC patients using real-time RT-PCR and identified a five-microRNA signature for the prediction of treatment outcome of NSCLC, which is an independent predictor of the cancer relapse and survival of NSCLC patients. Recently, Gao et al. [19] reported that seven-miRNA signature may have clinical implications in the outcome prediction of lung squamous cell carcinoma (LUSC). Of these 7 miRNAs, 2 (hsa-mir-139, hsa-mir-326) were negatively correlated with survival, while the other 5 (miR-101-2, miR-182, miR-183, miR-190, hsa-miR-944) were protective. Therefore, the identification of a miRNA signature that can predict the benefit from diagnostic and prognostic would be definitely helpful for the clinical decision and management of NSCLC in patients. However, it remains ambiguous whether miR-493-5p and its target can predict the clinical decisions of NSCLC.

In the present study, we first analyzed raw datasets of mRNA epression profiles from the GEO database to identify potential molecular markers of poor prognosis in NSCLC patients. We found that ITGB1 expression levels were significantly higher in NSCLC tumor specimens compared to non-neoplastic tissues. Indeed, FC and *P* < 0.01 screening for cluster analysis showed that NSCLC was a common denominator of two datasets and our NSCLC biopsies. ITGB1, as a direct target of miR-134, was reported to promote the occurrence and metastasis of lung cancer. ITGB1 was identified as an independent prognostic integrin marker associated with OS in NSCLC, which provided important clues to understanding the molecular mechanism of metastasis and contributing to the therapeutic treatment of lung cancer [20–23].

Next, to identify the miRNA targeting to ITGB1 and potential molecular mechanism, we used three target genes prediction websites to forecast target genes and validated that ITGB1 was a direct target of miR-493-5p using luciferase reporter assays. miR-493-5p was reported to can attenuate the invasiveness and tumorigenicity in human breast cancer. miR-493-5p is significantly up-regulated after imatinib treatment in chronic myeloid leukemia cell line K562 and over-expression of miR-493-5p can significantly inhibit K562 cellular growth, suggesting that miR-493-5p, as a tumor supressor factor, has therapeutic potential.

In this study, we confirmed for the first time that miR-493-5p expression in NSCLC samples was significantly lower compared to normal tissue, suggesting that down-regulation of miR-493-5p plays a crucial role in the progression of NSCLC. In order to explore the potential prognostic value of miR-493-5p, we analyzed the relationship between the expression of miR-493-5p and OS in NSCLC patients. Kaplan-Meier survival analysis showed that patients with low miR-493-5p expression levels had decreased OS. Furthermore, upregulated ITGB1 accompanied with downregulated miR-493-5p was associated with a shorter median OS. Thus, miR-493-5p and ITGB1 expression levels may be an optimal indicator and risk factor for reduced OS in NSCLC patients.

Taken together, our findings firstly indicate that miR-493-5p levels may play an essential role in NSCLC progression by targeting oncogene ITGB1 suggesting that ITGB1 and miR-493-5p have potential prognostic value as tumor biomarkers in NSCLC patients.

## MATERIALS AND METHODS

### Ethics statement

This study's protocol and acquisition of tissue specimens were approved by the Ethical Committee of Affiliated Hospital of Guangdong Medical University and Shanghai Tenth People's Hospital. Each participant provided written informed consent before participating in this study.

### Acquisition of clinical specimens

Fresh NSCLC samples were collected from patients undergoing surgical resection and classified according to the last WHO classification of lung cancers and clinical histories recorded, which was confirmed by two experienced pathologists independently. Demographic and clinicopathological characteristics were recorded including the patient's characteristics, tumor characteristics and overall survival time. The follow-up was conducted by telephone or direct correspondence. The time of tumor relapse or death was verified by the patient or their relatives, by medical recording, or by the social security record. Overall survival (OS) was calculated in months from the date of diagnosis to the time of death, regardless of cause.

### Bioinformatics analysis

The expression levels of mRNAs and miRNAs were investigated from GEO database (GSE41445, GSE67061-GPL6480, GSE61741, and GSE24709) (www.ncbi.nlm.nih.gov/geo/). Hierarchical clustering was performed using the multiple experiment viewer (MeV) 4.7.1 software programs: (https://sourceforge.net/projects/mev-tm4/files/mev-tm4/MeV%204.7.1/). We used three target-gene prediction software: RNA22-V2.0 (https://cm.jefferson.edu/rna22/), Targetscan (http://www.targetscan.org), and miRDB (http://mirdb.org/miRDB/) to forecast several potential target genes of miR-493-5p.

### Luciferase reporter assays

The human ITGB1 3′-UTR oligonucleotides containing the wild-type (Wt) or mutant (Mut) miR-493-5p binding site were sub-cloned into the *XhoI* and *NotI* sites of the pGL3 luciferase reporter plasmid vector (Promega, Madison, WI). For luciferase assay, HeLa cells were seeded in 24-well plates and cultured for 24 h; then, cells were co-transfected with either the Wt or Mut reporter plasmid. Forty-eight hours after transfection, luciferase assay was determined using the Dual-Luciferase kit (Promega, Madison, WI).

### RNA extraction and quantitative RT-PCR (qRT-PCR)

According to the manufacturer's guideline, the total RNA was isolated using Trizol regent (Invitrogen, Carlsbad, CA). RNA quantity was determined using NanoDrop ND-1000 spectrophotometer, the integrity of RNA was measured by gel electrophoresis.

For qRT-PCR, cDNA was synthesized from total RNA (10ng), and quantitative PCR reactions were performed with the Taqman Universal PCR Kit (Life Technologies). GAPDH and RNU6B were used as the internal control for ITGB1 and miR-493-5p, respectively. All the primers and probes of the ITGB1 and miR-493-5p, corresponding endogenous controls GAPDH and RNU6B for TaqMan miRNA assays were purchased from Applied Biosystems. The 2^-ΔΔCT^ method was used to analyze the expression levels.

### Western blotting

Total protein from HeLa cells on the condition of ITGB1 3′-UTR oligonucleotides containing the wild-type (Wt) or mutant (Mut) miR-493-5p mimics or inhibitor was extracted using cell lysis buffer to detect the expression levels of ITGB1 3′-UTR oligonucleotides containing the wild-type (Wt) or mutant (Mut) miR-493-5p and a reported target molecule FUT4. Protein concentration was analyzed using standard procedures for Western blotting. After incubation with the appropriate horseradish peroxidase-conjugated secondary antibodies, the membranes were treated with an enhanced chemiluminescence reagent (Thermo Scientific, Dreieich, Germany), exposed to X-ray film (Kodak, Rochester, USA) and quantified by densitometry (Beckman, South Pasadena, Canada).

### Cell lines culture and transfection

Human non-small cell lung cancer cell lineA549 were purchased from Shanghai Institute for Biological Sciences (Shanghai, China) and cultured in Dulbecco's modified Eagle's medium (DMEM; Gibco, USA) supplemented with 10% fetal bovine serum (FBS; HyClone, USA), 100 units/ml penicillin and 100 mg/ml streptomycin at 37°C in a humidified chamber supplemented with 5% CO_2_.

MiR-493-5p mimic and inhibitor were purchased from Life Technologies (TaqMan MicroRNA Assay). These molecular productions were transiently transfected into A549 cells, respectively, using Oligofectamin Transfection Reagent (Invitrogen) according to the manufacturer's instructions.

### Statistical analysis

The results were expressed as mean ± S.D. (standard deviation). Statistical comparisons between each group were conducted with Mann-Whitney U test or one-way ANOVA with the Kruskal-Wallis test. *P*-value less than 0.05 was considered statistically significant. Univariate survival analysis and multivariate analyses werecarried out using the Kaplan–Meier method. Spearman's correlation coefficient was used to test the relationship of expression level between miR-493-5p and ITGB1. All calculations were performed with the SPSS 20.0 software program (SPSS Inc, Chicago, IL, USA). The level of significance was chosen as *P* < 0.05.
